# AI-Driven Model for Automatic Emphysema Detection in Low-Dose Computed Tomography Using Disease-Specific Augmentation

**DOI:** 10.1007/s10278-022-00599-7

**Published:** 2022-02-18

**Authors:** Yeshaswini Nagaraj, Hendrik Joost Wisselink, Mieneke Rook, Jiali Cai, Sunil Belur Nagaraj, Grigory Sidorenkov, Raymond Veldhuis, Matthijs Oudkerk, Rozemarijn Vliegenthart, Peter van Ooijen

**Affiliations:** 1 Department of Radiation Oncology, University Medical Center Groningen, University of Groningen, Groningen, The Netherlands; 2 DASH, Machine Learning Lab, University Medical Center Groningen, University of Groningen, Groningen, The Netherlands; 3 Department of Radiology, University Medical Center Groningen, University of Groningen, Groningen, The Netherlands; 4 Department of Radiology, Martini Hospital Groningen, Groningen, The Netherlands; 5 Department of Epidemiology, University Medical Center Groningen, University of Groningen, Groningen, The Netherlands; 6 Department of Clinical Pharmacy and Pharmacology, University Medical Center Groningen, University of Groningen, Groningen, The Netherlands; 7 Faculty of Electrical Engineering, Mathematics Computer Science (EWI), Data Management Biometrics (DMB), University of Twente, Enschede, The Netherlands; 8 Faculty of Medical Sciences, University of Groningen, Groningen, The Netherlands; 9 Institute for DiagNostic Accuracy Research B.V., Groningen, The Netherlands

**Keywords:** Early diagnosis, Emphysema, Deep learning, Tomography, Minimum intensity projection

## Abstract

The objective of this study is to evaluate the feasibility of a disease-specific deep learning (DL) model based on minimum intensity projection (minIP) for automated emphysema detection in low-dose computed tomography (LDCT) scans. LDCT scans of 240 individuals from a population-based cohort in the Netherlands (ImaLife study, mean age ± SD = 57 ± 6 years) were retrospectively chosen for training and internal validation of the DL model. For independent testing, LDCT scans of 125 individuals from a lung cancer screening cohort in the USA (NLST study, mean age ± SD = 64 ± 5 years) were used. Dichotomous emphysema diagnosis based on radiologists’ annotation was used to develop the model. The automated model included minIP processing (slab thickness range: 1 mm to 11 mm), classification, and detection maps generation. The data-split for the pipeline evaluation involved class-balanced and imbalanced settings. The proposed DL pipeline showed the highest performance (area under receiver operating characteristics curve) for 11 mm slab thickness in both the balanced (ImaLife = 0.90 ± 0.05) and the imbalanced dataset (NLST = 0.77 ± 0.06). For ImaLife subcohort, the variation in minIP slab thickness from 1 to 11 mm increased the DL model’s sensitivity from 75 to 88% and decreased the number of false-negative predictions from 10 to 5. The minIP-based DL model can automatically detect emphysema in LDCTs. The performance of thicker minIP slabs was better than that of thinner slabs. LDCT can be leveraged for emphysema detection by applying disease specific augmentation.

**Supplementary Information:**

The online version contains supplementary material available at 10.1007/s10278-022-00599-7.

## Introduction

Chronic obstructive pulmonary disease (COPD) is among the leading causes of early deaths worldwide [1], and 70% of COPD is estimated to be under-diagnosed [2, 3]. Emphysema is a key component of COPD that is characterized by the destruction of lung parenchyma [4]. Usually, emphysema is diagnosed at the later stages of the disease’s progression and is itself an independent risk factor for lung cancer [5]. Therefore, early detection of emphysema is important.

Low-dose computed tomography (LDCT) has been shown to be capable of detecting lung cancer and also provides an opportunity to detect comorbidities like emphysema in early stages [6]. However, LDCT contains inherent noise, and screening asymptomatic participants means there are more normal scans than abnormal ones, making emphysema detection labor-intensive [7, 8]. On CT imaging, emphysematous lung regions with reduced tissue density appear as areas of low attenuation. For quantitatively assessing emphysema in CT, the low attenuation areas (LAA) under a specific cut-off threshold value are computed, for example, less than −950 HU. Although this method is widely used, it is prone to measurement variation and lacks consensus on an optimal cut-off threshold, leading to uncertainty in the diagnosis [9]. Past studies have proposed automatic emphysema detection using deep learning (DL) algorithms as a solution to bypass these issues and reduce the burden on radiologists while leveraging the lung cancer screening LDCT dataset [10, 11].

The existing supervised machine learning algorithms [12, 13] or DL algorithms for automatic emphysema detection like 3D convolutional neural networks (CNNs) [14], deep-CNNs with long short-term memory [15], and transfer learning models like 3D ResNet [16] require disease localized annotations, which are difficult to obtain for large datasets, or they are primarily developed using HRCT [17, 18]. This motivated us to develop an unsupervised model for emphysema in screening studies.

Data augmentations have been suggested as an aid to task-specific unsupervised DL models [19]. Typically, data augmentation consists of techniques such as geometric transformation, kernel filters, and feature augmentation that enhance the size and quality of the training dataset for the task-specific models [20].

In emphysema diagnostics, minimum intensity projection (minIP) is used as a visualization technique to detect low-density structures (low attenuation areas) in a given computed tomography (CT) volume and emphasize the subtle features of trace and mild emphysema [21, 22]. The purpose of this study is to test the feasibility of applying minIP as a disease-specific augmentation to the proposed unsupervised DL algorithm for automatic emphysema detection in LDCT.

Studies have indicated that varying minIP slab thickness can affect the qualitative assessment of emphysema [23, 24]. However, its effects on DL models remain unknown, and so, along with the development of a minIP-based DL algorithm, we investigated the effects of different slab thicknesses on the DL algorithm for emphysema.

The notable contributions of this study are as follows: (1) we used an unsupervised DL algorithm to address the annotation-less and class-imbalanced scenarios that normally characterize lung cancer screening LDCTs; (2) we tested the feasibility of using clinical domain knowledge such as minIP to emphasize the minimal differences of emphysema regions in LDCT for unsupervised learning; (3) we explored the effects of different slab thickness of minIP on DL algorithm; (4) we generated detection maps to interpret the model predictions and to serve as a quality check; and (5) we validated our model on lung cancer screening data to check the efficacy of the proposed DL algorithm in a real use-case.

## Materials and Method

### Study Population

Medical ethics committee approval was obtained prior to the study. The population for this retrospective study was chosen from two different cohorts. The first was a general population study in the Netherlands (Imaging in Lifelines or ImaLife) designed to find early imaging biomarkers for the “big-three” thoracic diseases, COPD, coronary artery disease, and lung cancer. The second was the National Lung Cancer Screening Trial (NLST) which was carried in the USA. It is one of the biggest collections of lung cancer screening LDCT data and contains case-specific datasets with annotations. The details regarding the eligibility criteria for the participants of ImaLife and NLST have been previously described [25, 26].

### Acquisition Protocol

The CT acquisition protocol for the ImaLife study participants included a low-dose chest CT examination with a third-generation dual-source CT scanner (SOMATOM Force, Siemens). All the scans were reconstructed in the axial plane with filtered back projection using a soft kernel (Br40) with a single slice thickness of 1 mm and 0.7-mm increments [25]. The NLST subcohort contained LDCT scans from various hospitals where multi-detector scanners from GE medical systems, Siemens, Toshiba, and Philips with varying slice-thicknesses were used to produce the scans [27]. A brief overview of CT acquisition and reconstruction protocols used in the subcohorts is shown in Table 1. All the scans used in this study were acquired during end-inspiration breath-hold and without the administration of any contrast media.

**Table 1:** The CT acquisition and reconstruction protocol for the dataset from ImaLife and NLST

Acquisition parameters	ImaLife	NLST
Slice thickness (mm)	1	1.0–3.2
Slice increment (mm)	0.7	1.0–2.5
Scan mode	High pitch spiral	Helical CT
Pitch	3.0/2.5	0.8–1.5
Tube voltage (kVp)	120	120
Tube current (mAs)	20	40–120
API	Inspiration breath-hold	Inspiratory breath-hold
Window width (HU)	350	400
Window level	50	40
Reconstruction filter	Br40	Standard, B30f, FC51, B50f, FC30

### Visual Scoring

Each scan from ImaLife was visually scored by one of three trained medical professionals (two radiologists with more than 10 years of experience and one trained technical physician). The three readers followed a standard annotation protocol based on the Fleischner criteria [28]. For this project, the visual scoring was consolidated to dichotomous emphysema diagnosis per scan. The scans with no emphysema annotation were considered normal, and the severity categories trace, mild, moderate, confluent, and advanced destructive emphysema were considered abnormal. For NLST subcohort, radiologists from various hospitals participating in the NLST screening trial had annotated the scans with a yes or no diagnosis for emphysema, and this information was directly used for the external validation [27].

### Quantitative CT Measurements of Emphysema

To measure the quantitative characteristics of the subcohorts and evaluate the potential of DL algorithm trained on visual scoring over traditional quantitative analysis, we performed quantitative CT measurements of emphysema using the routinely used automatic densitometry tool (version 4.4.13, Aquarius iNtuition, TeraRecon). The lung region was semi-automatically segmented, and emphysema was quantified as the percentage of all lung area (voxels) having attenuation lower than −950HU (%LAA). Participants with %LAA were divided into two subgroups for analysis, with %LAA ≤ 5% categorized as non-emphysema and %LAA > 5% categorized as emphysema [29].

### Minimum Intensity Projection (minIP)

MinIP is a volume rendering technique where the voxels with lowest attenuation in an image slice are projected to form a bidimensional slab. The slab thickness can be varied based on the number of slices used. First, the acquired scans were re-sampled and normalized to compensate for kernel differences [30]. Then the voxels with lowest Hounsfield units on each slice of a patient scan were projected to form varying slab-thickness. In this study, we generated minIP slabs with thicknesses ranging from 1 up to 11 mm in 2-mm increments for the comparative evaluation. In routine evaluation for emphysema, radiologists use 5 to 10 mm minIP slabs on every slices to carefully check for low attenuation areas [21, 31]. Our study evaluated a wider range to assess the effectiveness of the DL algorithm for each slab thickness separately. Illustration of emphysema case with different MinIP settings with slab thicknesses varying between 1 and 100 mm is shown in Fig. A of Online Resource 1. The algorithm for minIP is implemented in Python and will be made available upon request.

### Training and Testing Split

**Fig. 1:**
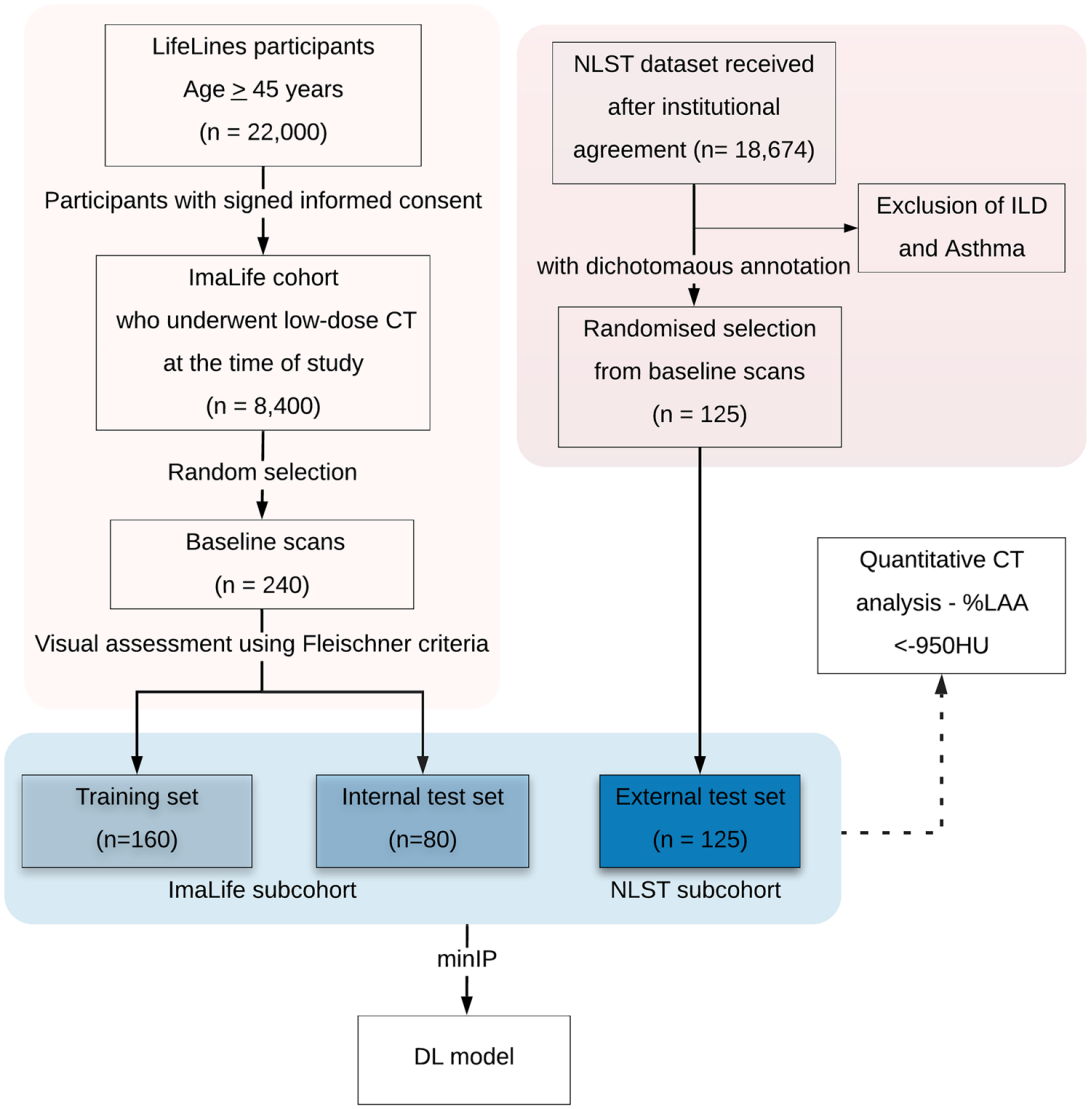
The flowchart indicates the inclusion criteria and the data split for the training and internal and external datasets in the study. All the scans used for current study are the baseline or earliest scan available for each participant. The quantitative CT analysis involved measuring percentage low attenuation areas < −950HU on all three datasets

For the development of the DL model, the subcohorts were divided into three datasets. The first was a dataset containing non-emphysema scans for adversarial auto-encoder training, the second was a class-balanced dataset for internal validation, and the third was a class-imbalanced (higher number of non-emphysema scans) dataset for external validation. A total of 160 (80%) out of 200 non-emphysema scans from the ImaLife subcohort were used as the training dataset. The internal validation dataset contained the remaining 40 (20%) normal scans and 40 emphysema scans from the ImaLife subcohort. The complete NLST subcohort was used as the external validation dataset. The consort diagram illustrating the data streams for training and internal and external validation is shown in Fig. 1. It is important to note that the above procedure was followed separately for each minIP slab thickness.

### Deep Learning Algorithm

To build a prototype for automatic classification of emphysema in an annotation-less, class-imbalanced environment, we used an unsupervised anomaly detection scheme. The model takes minIPs of certain slab thickness as inputs with a dimension of (512 × 512) × *n* sampled evenly over the height of the lungs, where *n* represents the number of axial slices for each participant. The predictions are then concatenated in a sequence of axial slices for the final participant score. The model performs classification on the participant level, so that for a participant to be classified as negative, all slices considered from each participant scan had to be classified as non-emphysema. Otherwise, the participant was classified as positive. The complete DL algorithm was implemented on PyTorch (version 5.1, Python 3.7.1, CUDA 10.1) and executed on a NVIDIA Titan XP GPU with 16 GB memory.

### Model Architecture

**Fig. 2:**
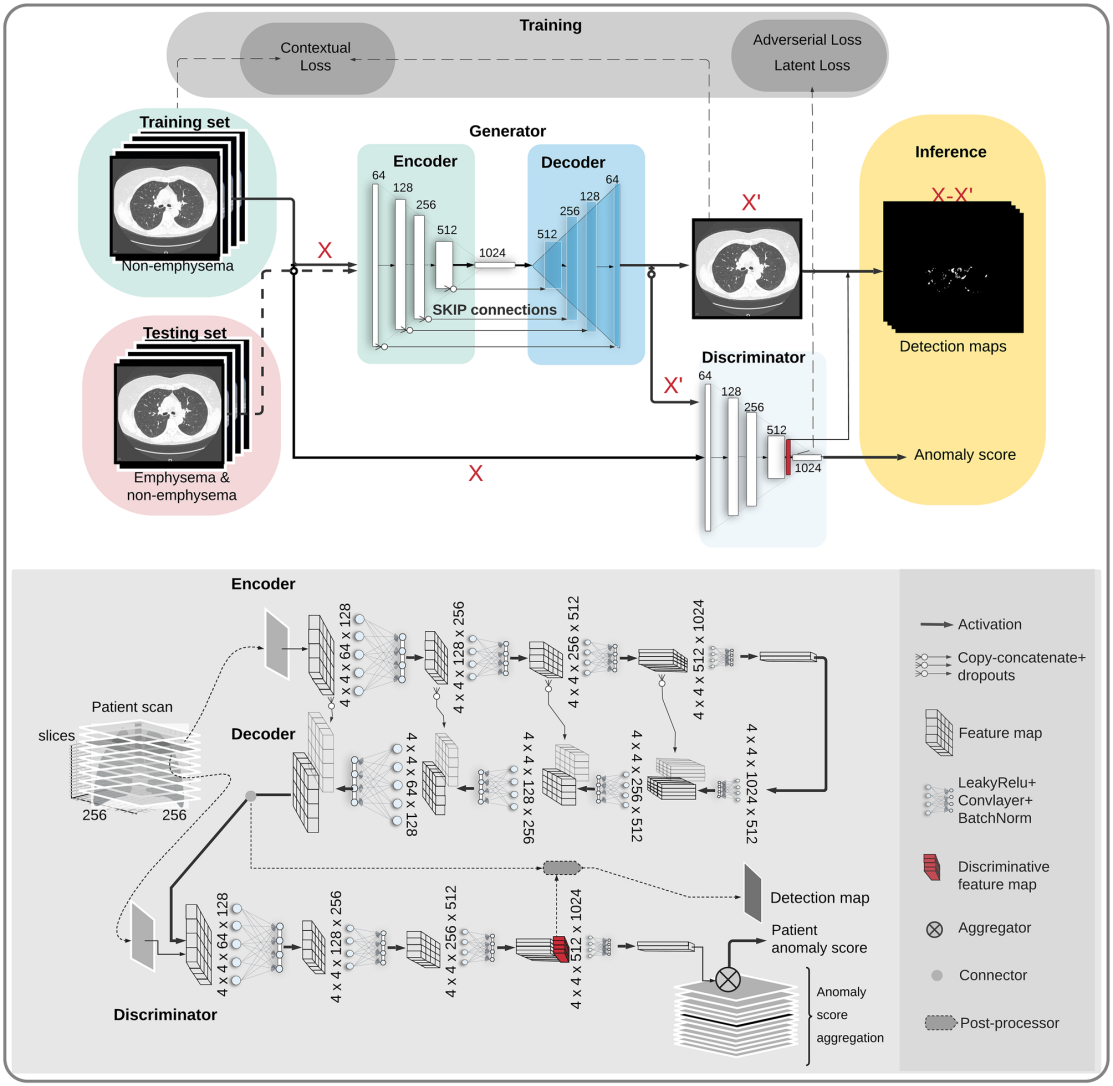
The workflow of adversarial architecture for automatic emphysema classification and detection in LDCT is shown in the top figure. The generator consists of encoder and decoder blocks with 8 layers of 4 × 4 kernel size and stride 2. The layers are connected to each other over short-ranged connection and long ranged skip connections. The discriminator architecture is similar to the encoder architecture. The combined learning (training) of generator and discriminator happens by minimizing the loss functions. The discriminator is a feature extractor which can extract features within the latent space and a classifier that provides prediction score and detection maps during inference. The bottom figure shows the properties of each layer and are indicated with four hyperparameters in this order: first dimension of the kernel × the second dimension of the kernel × the number of input channels × the number of output channels at each convolution

The classification network was built using adversarial auto-encoders, which can be divided into a generator block and a discriminator block (Fig. 2). The first part of the generator block consists of an encoder with fully convolutional layers capable of encoding the high-dimensional image-representation into a low-dimensional latent-representation. The second part of the generator architecture is a decoder that can decode the low-dimensional latent-representation back into a high-dimensional image-representation (X’) [32]. The generator architecture includes sixteen fully convolutional downsampling and upsampling layers with a kernel size of 4 × 4 and stride 2, where each layer is followed by batch normalization and Leaky ReLU activation functions. The details of architectural components and the loss functions have been defined earlier [33]. The encoder’s feature maps are forwarded to the decoder via skip connections with dropout regularization to translate intrinsic image information between blocks without overfitting. This feature maps from the decoder are fed to the discriminator block and the prediction outputs are obtained as anomaly score for each participant scan. The discriminator block has a similar architecture as the generator’s encoder, consisting of eight fully convolutional downsampling layers with a kernel size of 4 × 4 and stride 2, where each layer is followed by batch normalization and Leaky ReLU activation function. We used the discriminative image features from the last convolutional layer of the discriminator as a priori to decide the candidate/anomaly regions in detection maps. Additionally, to train the model with a limited dataset and avoid overfitting, we incorporated discriminator heuristics augmentation [34].

### Model Training

During training, the model was fed with axial slices of given minIP slab thickness in which emphysema was not present so that the generator and the discriminator combination could learn to map the intrinsic properties of non-emphysema lungs. The training was based on minimizing the three loss functions, namely, contextual loss based on L1 distance (or generator’s loss), latent loss (L2 distance measure), and adversarial loss (or discriminator’s loss). The definitions of these three loss functions have been previously published [32].

The trained network in the inference phase classifies the abnormal emphysema scans as anomalies and calculates a prediction score based on generator loss and latent loss functions. The prediction score is the prediction probability provided by the discriminator as a continuous variable ranging from 0 to 1, with higher scores corresponding to emphysema detected.

### Detection Maps

Along with the prediction scores, the modified network was designed to provide binary masks or residual images for every test image. This was done by subtracting the input image (X) and the generated image (X’) from the generator block [35]. Afterwards, these residual images were post-processed using a lung lobe segmentation algorithm to automatically remove anything apart from the candidate regions generating detection maps. We used an available pre-trained deep learning algorithm trained on the Lung Tissue Research Consortium dataset to perform the post-processing task [36]. The detection maps were then superimposed on the input image to serve as a quality check.

### Model Evaluation and Statistics

Our model evaluation was performed in three different stages:

- Stage 1: Internal–external validation: The validation findings for each minIP slab thickness (1, 3, 5, 7, 9, and 11 mm) were analyzed using the model’s area under the receiver operating curve (AUC), sensitivity, and specificity. Bootstrapping with 1,000 iterations was performed on AUC to find the confidence interval (CI). Since the external validation dataset was chosen to be a class-imbalanced dataset, we incorporated the F1 score as one of the performance indicators [37]. The optimal minIP slab thickness must show high sensitivity and AUC, with a low number of false negatives. To compare the performances of the various minIP slab thickness, we used McNemar’s test in the IBM SPSS Statistics tool (version 22) [38].
- Stage 2: The inter-rater reliability between visual scoring and %LAA was assessed using Kappa. In addition, to test the performance of the visual scoring-based DL algorithm on %LAA-based categorization, we used our optimal minIP slab thickness.
- Stage 3: To enhance the explainability of the DL algorithm, randomly selected 2D detection maps from the DL algorithm were compared to the bounding box annotations of the radiologists.

## Results

### Population Characteristics

The ImaLife subcohort used for training and internal validation consisted of 240 participants (male = 116 [48.4%] and female = 124 [51.6%]); the mean age ± SD at enrollment was 57 ± 6 years. Visual scoring by radiologists indicated 40 (17%) individuals with emphysema in the subcohort. The quantitative CT measurement of emphysema (%LAA −950) in the ImaLife subcohort showed a median of 4% with an interquartile range of 1.8 to 7.8%. Out of 240 individuals, 136 (56.6%) had %LAA ≤ 5%, and 104 (43.4%) had %LAA > 5%.

The NLST subcohort contained 125 (male = 79 [63.2%] and female = 46 [36.8%]) patients with mean age ± SD of 64 ± 5 years at enrollment. The emphysema annotation of the NLST subcohort indicated that 33% individuals had emphysema. The quantitative CT measurement subcohort had median of 15.1% (interquartile range, 5.3 to 28.3%) of emphysema. Out of 125 patients, 25 (20%) had %LAA ≤ 5% and 100 (80%) had %LAA > 5%.

The population characteristics of the subcohorts are shown in Table 2.

**Table 2:** Population characteristics of ImaLife and NLST subcohorts

Parameters	ImaLife (*n* = 240)	NLST (*n* = 125)
Age	56.6 ± 6.2	64.5 ± 5.4
Sex		
Male	116 (48.3%)	79 (63.2%)
Female	124 (51.7%)	46 (36.8%)
Visual emphysema scoring		
Non-emphysema	200 (83%)	83 (66.4%)
Emphysema	40 (17%)	42 (33.6%)
Trace	8 (20.0%)	-
Mild	16 (40%)	-
Moderate	11 (27.5%)	-
Confluent	4 (10%)	-
Advanced destruction	1 (2.5%)	-
Quantitative CT analysis		
Non-emphysema (%LAA ≤ 5%)	136 (56.6%)	25 (20.0%)
Emphysema (%LAA > 5%)	104 (43.4%)	100 (80.0%)

### Model Evaluation

#### Internal Validation

**Fig. 3:**
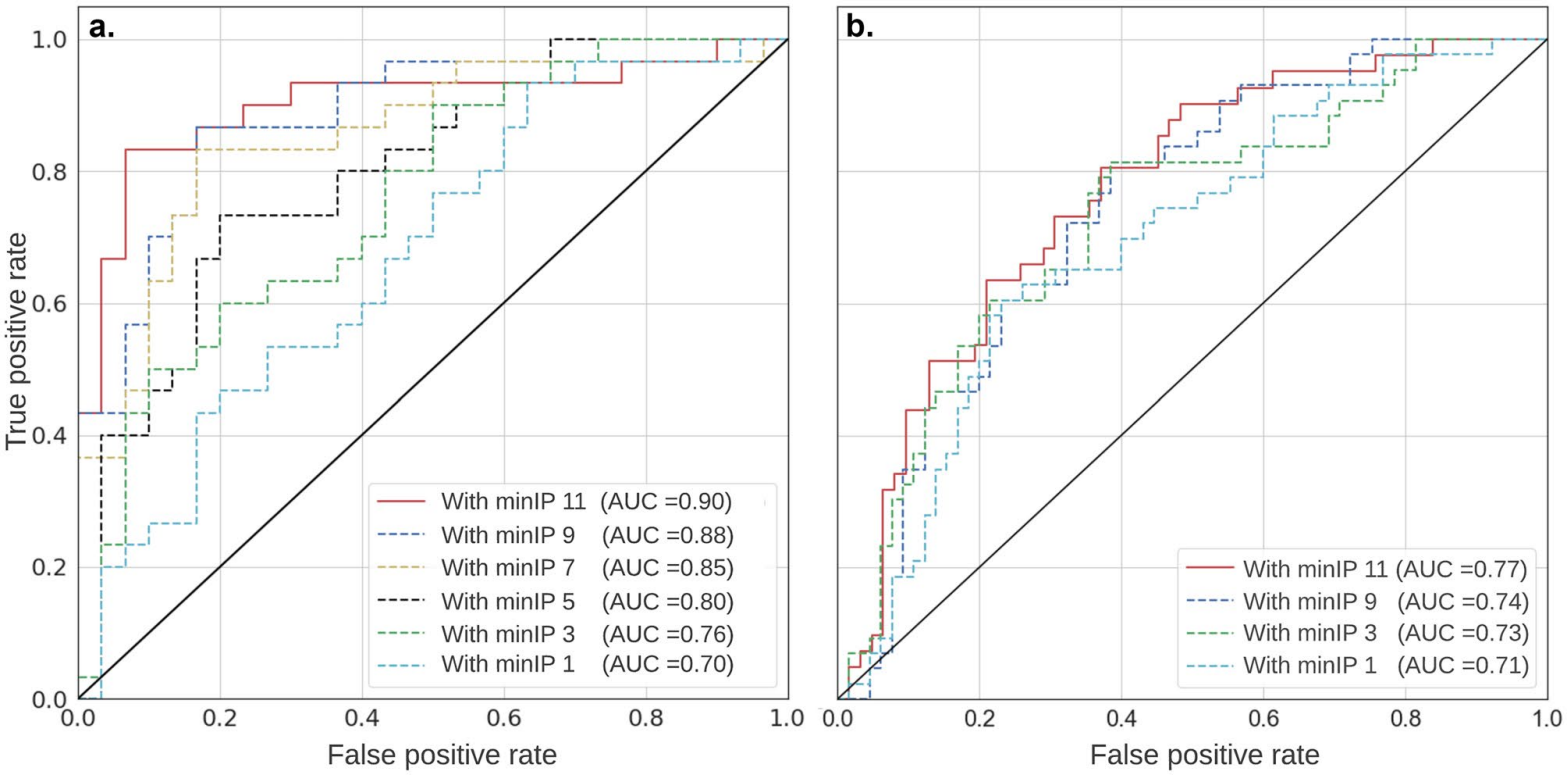
The area under the curve obtained for the proposed DL model with different minIP slab thicknesses. **a**. ImaLife subcohort, **b**. NLST subcohort. Note that 11 mm slab thickness yielded the highest AUC

The minIP-based DL model automatically detected emphysema in the ImaLife subcohort with a sensitivity of 88% in a class-balanced setting. The internal validation results are shown in Fig. 3a and Table 3. For the slab thicknesses from 1 to 11 mm, there was a positive effect on the DL pipeline performance, that is, increasing the slab thickness resulted in an increase in area under receiver operating characteristics curve (AUC). The classifier’s false-negative predictions decreased by 50% (from 10 to 5) when the slab thickness was increased (1 to 11 mm). Of all the minIP slab thicknesses that were tested, the DL pipeline showed the highest performance for 9 and 11 mm, both in terms of AUC (0.90 ± 0.05 and 0.88 ± 0.05) and false negatives (5/40 and 6/40). There was a small but statistically significant difference of 2% in AUC between 9 and 11 mm (*p*-value = 0.0348).

**Table 3:** Performance metrics of the DL model for different minIP slab-thicknesses on the ImaLife subcohort

Setting	AUC	Sensitivity	Specificity	False-negative	False-positive	F1 score
minIP 11	0.90 ± 0.05	0.88 ± 0.05	0.83 ± 0.06	5/40	7/40	0.85
minIP 9	0.88 ± 0.05	0.85 ± 0.04	0.85 ± 0.07	6/40	6/40	0.85
minIP 7	0.85 ± 0.06	0.83 ± 0.05	0.85 ± 0.06	7/40	6/40	0.84
minIP 5	0.80 ± 0.05	0.83 ± 0.03	0.80 ± 0.05	7/40	8/40	0.81
minIP 3	0.76 ± 0.05	0.77 ± 0.07	0.83 ± 0.04	9/40	7/40	0.79
minIP 1	0.70 ± 0.07	0.75 ± 0.05	0.87 ± 0.08	10/40	5/40	0.80

*minIP* minimum intensity projection, *AUC* area under the curve

**Fig. 4:**
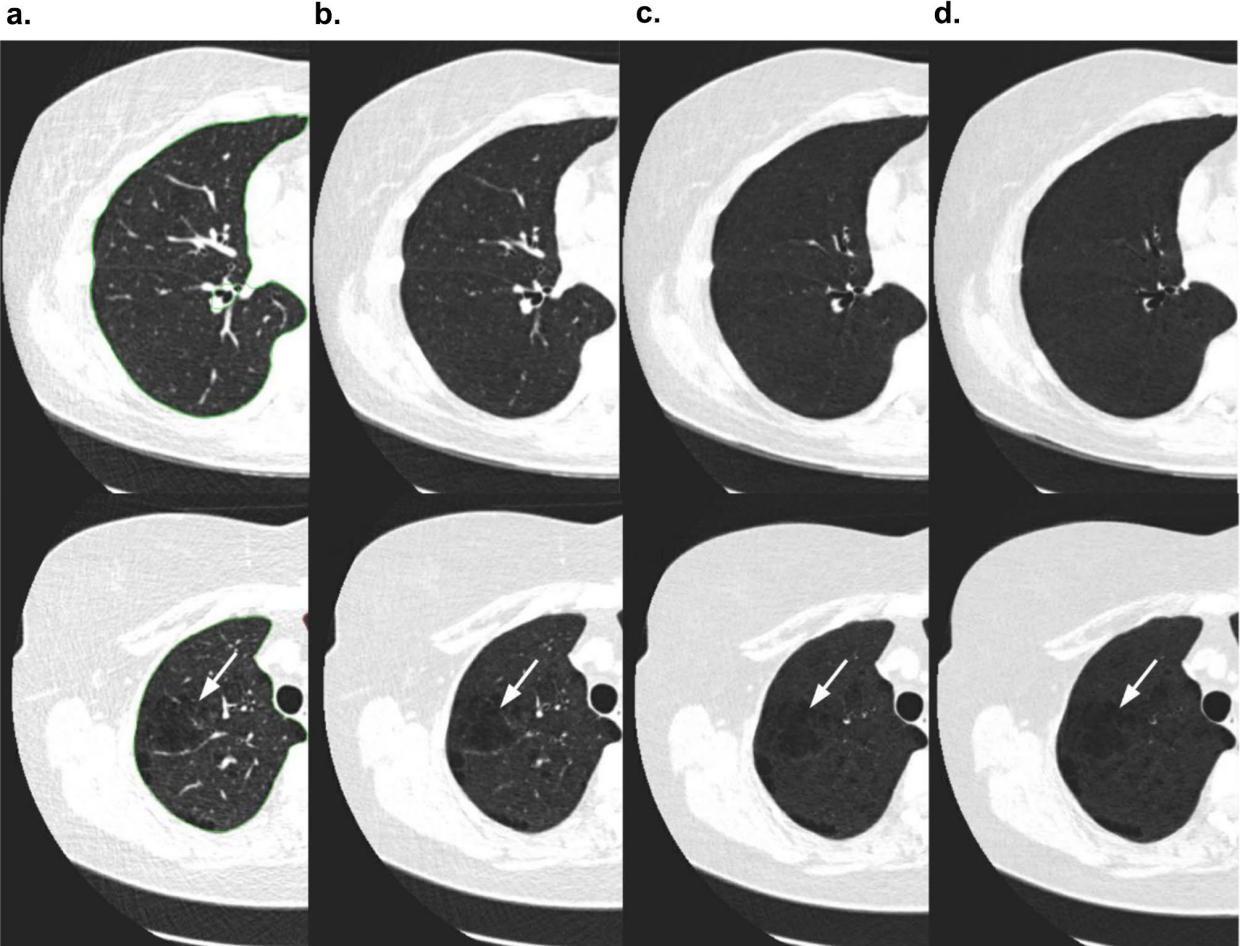
Application of various minimum intensity projection slab thicknesses on thin-section CT obtained at the same anatomic level and magnified views of the lung (window width, 500 HU; window level, -850 HU). **a**. Thin-section CT scan (1 mm-collimation) and **b**, **c** and **d** minimum intensity projection images with 3 mm, 7 mm, and 11 mm collimation. The first row represents a 52-year-old participant with non-emphysema diagnosis and the bottom row represents an emphysema participant of age 60 years. Note progressive suppression of vascular structures from 3 to 7 mm slab thickness, and better visualization low attenuation areas (white arrow) (For interpretation of the reference to color in the figure legend, the reader is referred to the web version of the article)

In Fig. 4, examples of non-emphysema and emphysema scans before and after applying minIP are shown. It can be seen that apart from helping to visualize low-density regions, or emphysema regions, applying minIP also reduces the complexity of the image by suppressing the high-contrast regions. Our DL algorithm was run without any minIP, which clearly demonstrated that adding greater than 1 mm minIP slab thickness to 1 mm slice thickness LDCT scans can improve the algorithm’s performance. This is shown using the class separation plots obtained from the DL algorithm for emphysema and non-emphysema scans in Fig. B of Online Resource 1 (electronic supplementary material), where an increase in separation between the classes is observed as the minIP slab thickness increases.

#### External Validation

In the NLST subcohort out of 33% emphysema scans, 79% were detected by the DL pipeline. In a class-imbalanced setting, the 11 mm slab thickness was found to be most sensitive, with a performance of AUC = 0.77 ± 0.06 (Fig. 3b) and nine false negatives being the least among all the minIP slabs (Table 4). The model’s F1 scores were highest for 11 mm (0.70) and 9 mm (0.67) slab thickness, indicating an acceptable performance in a real-world setting.

**Table 4:** Performance of the DL model for different minIP slab-thicknesses on the NLST subcohort

Setting	AUC	Sensitivity	Specificity	False-negative	False-positive	F1 score
minIP 11	0.77 ± 0.06	0.79 ± 0.05	0.77 ± 0.06	9/42	19/83	0.70
minIP 9	0.74 ± 0.06	0.76 ± 0.04	0.74 ± 0.07	10/42	21/83	0.67
minIP 7	0.67 ± 0.08	0.67 ± 0.05	0.73 ± 0.06	14/42	22/83	0.62
minIP 5	0.74 ± 0.05	0.71 ± 0.03	0.75 ± 0.05	12/42	21/83	0.65
minIP 3	0.73 ± 0.03	0.74 ± 0.07	0.72 ± 0.04	11/42	23/83	0.65
minIP 1	0.71 ± 0.03	0.69 ± 0.05	0.74 ± 0.08	13/42	21/83	0.63

*minIP* minimum intensity projection, *AUC* area under the curve

#### Quantitative CT-Based Evaluation

In the ImaLife subcohort, there was fair agreement on emphysema and non-emphysema scans between our center’s radiologists and quantitative CT analysis (63% concordance, kappa 0.241). In the NLST subcohort, a slight agreement between procured annotation and quantitative CT analysis with 40% concordance (kappa 0.104) was observed. This indicated that the label noise in the NLST subcohort was higher than that of the ImaLife subcohort.

Our optimal minIP model (minIP slab thickness 11 mm), when tested on %LAA-based categorization, yielded an AUC of 0.79 ± 0.02 in the ImaLife subcohort and an AUC of 0.70 ± 0.04 in the NLST subcohort.

### Detection Maps

**Fig. 5:**
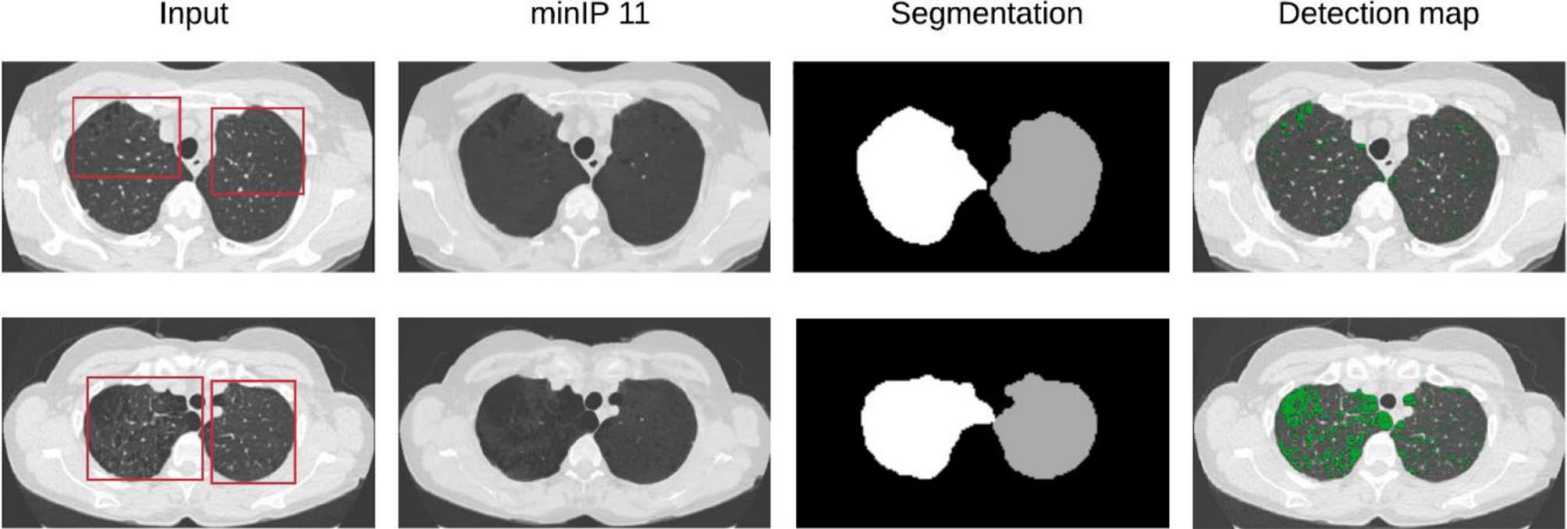
Explainability of the deep learning model. Randomly selected abnormal images (axial-emphysema scans) which were correctly classified by the model are illustrated here. The first column shows the example images with radiologist annotation using red bounding box. The bounding box’s in these images were only used to illustrate the emphysema regions. The following column represents the corresponding minIP images of slab thickness 11. Third and fourth column illustrates the lobe segmentation masks (color-coded as white for left lobe and grey for right lobe) followed by detection maps from the DL model, respectively. The green regions in the detection maps represent the detected emphysema regions. The detection maps indicated the presence of emphysema inside the bounding box provided by radiologists (For interpretation of the reference to color in the figure legend, the reader is referred to the web version of the article)

The quality check of the detection maps from the DL model by the radiologists revealed that the prediction regions in detection maps were accurate in highlighting emphysema regions in 97% (34 out of 35) and 91% (30 out of 33) of the predicted cases in internal and external-validation datasets, respectively. An example of a detection map is illustrated in Fig. 5.

## Discussion

This novel study aimed to evaluate the feasibility of a minIP-based DL algorithm for automatic emphysema detection in LDCT. Our results show that the proposed DL model trained on LDCT accurately detects the presence of emphysema with a sensitivity of 88% and aids emphysema detection in lung cancer screening. The multi-scale assessment of the DL model revealed that 11 mm minIP slab thickness was optimal in both general population (90%) and lung cancer screening (77%) datasets. The application of minIP enabled the unsupervised DL model to learn faster (in fewer epochs) by reducing the complexity of the image (suppressing the vessels and high-intensity phenotypes) and enabling the DL model to focus on disease-specific features. MinIP also helped collapse 3D information into a more efficient 2D representation, thereby reducing the computational burden. In our study, the performance of thicker minIP slabs (7 to 11 mm) was better than that of thinner slabs (1 to 3 mm). This is similar to those used in the routine clinical evaluation. Lan et al. also found thick slab minIPs (5 to 10 mm) to be more effective, with negligible differences between these minIP slabs [22].

**Fig. 6:**
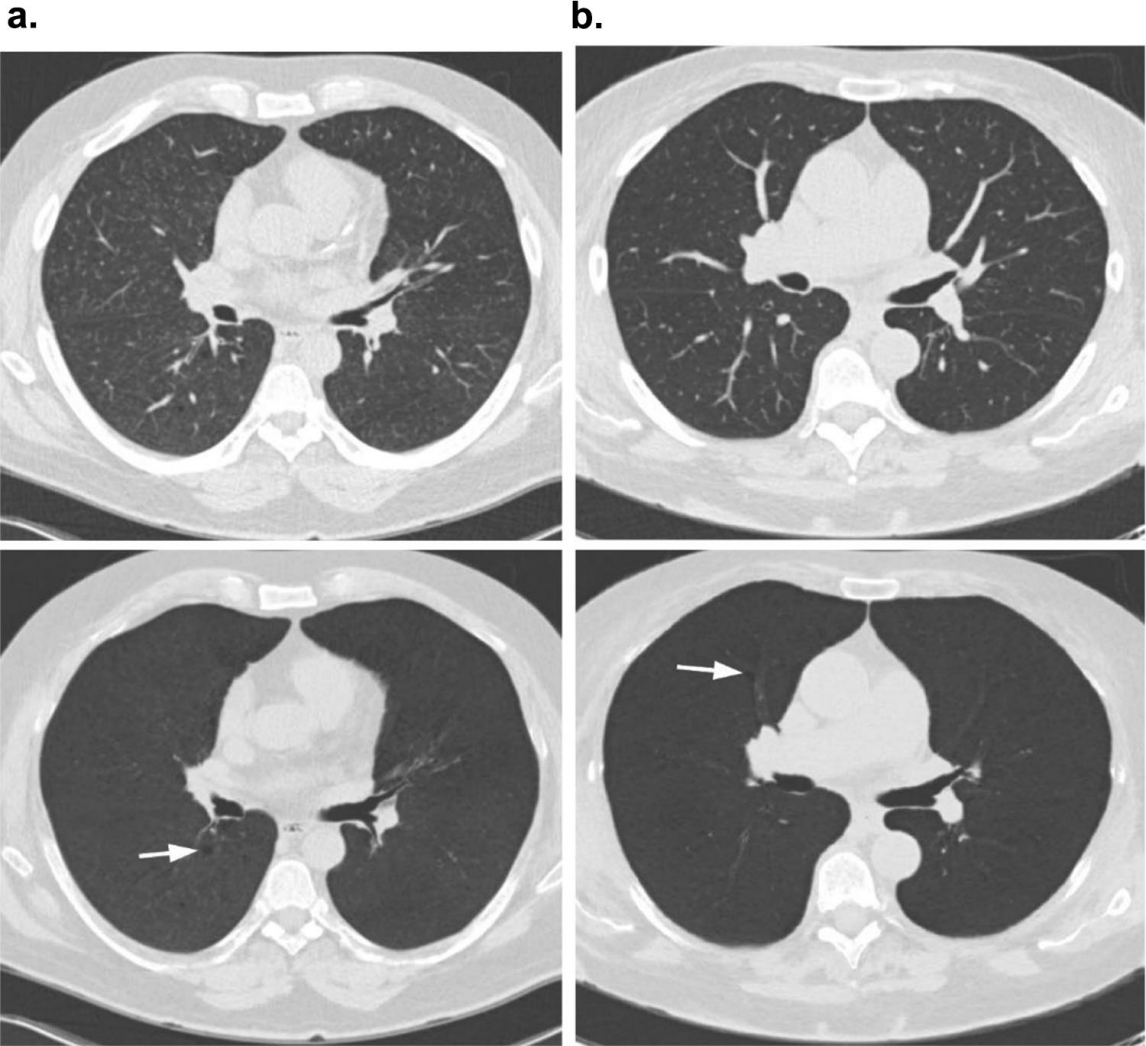
Examples of false negative scans. First row represents multi-planar reconstruction (MPR) visualization and second row represents minimum intensity projection of the corresponding MPR images. **a**. 56 years male with smoking history of 45 pack years. **b**. 65 years female with smoking history of 40 pack years. The white arrows indicate the Fleischner criteria defined traces of emphysema in the lung that was missed by the DL model

The external validation of the DL model on the class-imbalanced NLST subcohort achieved a sensitivity of 79%, and there was a drop in overall model performance compared to the internal validation. This might be for two reasons. The first is the label noise: in the NLST dataset, the presence of emphysema on imaging is only recorded as yes or no and does not indicate any additional information on the evaluation method or protocol used, making meaningful interpretation difficult. The second reason is that the model is sensitive to reconstruction parameters (especially slice thickness and slice increment) and the NLST dataset contained varying reconstruction parameters, which could have influenced the model’s performance. Although the variation in slice thickness in the external validation dataset could have been compensated for, the scope of the study was to test the model’s constraints and check its creditability in a real-world setting. Examples of false negative cases that the model failed to classify are shown in Fig. 6.

In the clinical setting, lung densitometry (%LAA) is predominantly used to assess emphysema in CT. However, visual emphysema scoring is less sensitive to image noise and can more precisely discriminate between the presence or absence of emphysema [39] and so, our model was developed on visual scoring instead of CT densitometry based scoring. Concurrently, we found the kappa agreement between visual scoring and CT densitometry to be fair and slight for ImaLife and NLST subcohorts, respectively. A similar level of agreement was observed between visual assessment and quantitative CT in the COPDGene study for emphysema detection [39].

Tang et al. developed a transfer learning DL pipeline to classify COPD patients in lung cancer screening LDCTs. Although the authors do not address emphysema specifically, the reported AUC of the model on percentage low attenuation areas (%LAA) was 74% [11]. Therefore, we tested our DL model on %LAA-based categorization and found comparable results.

Furthermore, Humphries et al. performed automatic classification of emphysema using a CNN-LSTM network with 79% accuracy on COPDGene datasets [15], and Hatt et al. used a dataset from a similar cohort using a 3D-Resnet-CNN model and achieved an accuracy of 79.8% [16]. Although these studies have shown that it is possible to use DL models for emphysema, they vary in terms of model architecture (supervised) and inclusion protocol of participants (based on CT dose, GOLD criteria, or scoring), and so directly comparing them with our model is not possible. Moreover, none of the studies addresses the data imbalance that exists in the real-world scenario, while our model with adversarial training is well-suited to class-imbalanced tasks. Previously Nagaraj et al. compared the same adversarial network with RESNET in class-imbalance settings and adversarial model outperformed the pre-trained model showing the potential of adversarial network [33].

In this study, detection maps for anomalies were compared to the visual identification of emphysema by radiologists, and clinically acceptable performance was observed. Although the pixel-wise emphysema localization via detection maps can be used to verify the model’s predictions, the detection maps are only 2D axial sections, and they cannot be considered for 3D emphysema quantification. However, they may be used as an annotation tool for emphysema.

For our future work, we intend to combine objective measurements such as pre- and post-bronchodilator spirometry combined with visual scoring to validate the classifier’s performance. By utilizing our model, the radiologists’ confidence in identifying emphysema can be increased by providing a comparison methodology like our model that is capable of classifying the overall scans into emphysema and non-emphysema categories and pinpointing the emphysema regions in the scans. Additionally, there is no threshold to toggle as in the HU-based method, which is usually subject to bias.

The main limitation of our model is that it does not classify emphysema based on severity levels. Our immediate future work will focus on training the minIP model with a multi-protocol diverse dataset. Additionally, the experiments will be designed to evaluate the effects of combining multiple minIPs to determine whether this can compensate for protocol variations and validate the model on a large scale lung cancer screening dataset. Combining this DL model with automatic nodule exclusion may aid comprehensive lung disease and mortality evaluation in LDCT lung cancer screening.

## Conclusions

We developed an automatic minIP-based DL model for the classification and detection of emphysema in LDCT. Using minIP as a disease-specific augmentation technique, the unsupervised DL algorithm becomes a robust model to address the annotation-less and class-imbalanced scenarios that normally characterize lung cancer screening LDCTs. The DL model is sensitive to scan slice thickness and needs to be validated on multi-cohort datasets. When deployed in screening setup, our model can assist large-scale emphysema classification and provide the detection maps that can act as priori to increase the confidence in the decision.

## Supplementary Information

Below is the link to the electronic supplementary material.

Supplementary file1 (PDF 251 KB)

## Data Availability

NLST data is available on request.

## Abbreviations and Acronyms

minIP: Minimum intensity projection
DL: Deep learning
ImaLife: Imaging in lifelines
NLST: National lung cancer screening trail
LDCT: Low-dose computer tomography
HU: Hounsfield unit
LAA: Low attenuation areas
AUC: Area under the receiver operating curve
CI: Confidence interval

## Code Availability

Available on request.

## Declarations

### Study Subjects or Cohorts Overlap

The details of eligibility criteria for the participants of ImaLife and National Lung Cancer screening (NLST) have been previously described. There was no-overlap of study subjects or cohorts.

### Statistics and Biometry

One of the authors has significant statistical expertise.
