# Supplementary material for: AI-Driven Model for Automatic Emphysema Detection in Low-Dose Computed Tomography Using Disease-Specific Augmentation
Source: J Digit Imaging. 2022 Feb 18;35(3):538–50. doi: 10.1007/s10278-022-00599-7 (PMC9156637; doi:10.1007/s10278-022-00599-7)
Supplement: Supplementary file 1 — Supplementary file1 (PDF 251 KB) [file 10278_2022_599_MOESM1_ESM.pdf]

Electronic supplementary material

Online Resource 1: **Higher minIP settings**

In the clinical setting, radiologists use minIP slab thickness between 5 mm to 10 mm. Though minIP is well suited to suppress the anatomic background (maximum intensity values like vessels) and display emphysema regions in the lung parenchyma. A very high minIP slab thickness might clip-off the lung structures. For example, in the second row of the figure below, the emphysema region (red arrow) around airways regions merge with bronchi with increasing slab-thickness. This also answers the question of our choice of the range of the slab thickness for the current study. We think that the loss of anatomical information of the lung due to higher minIP can lead to false interpretations of DL model.

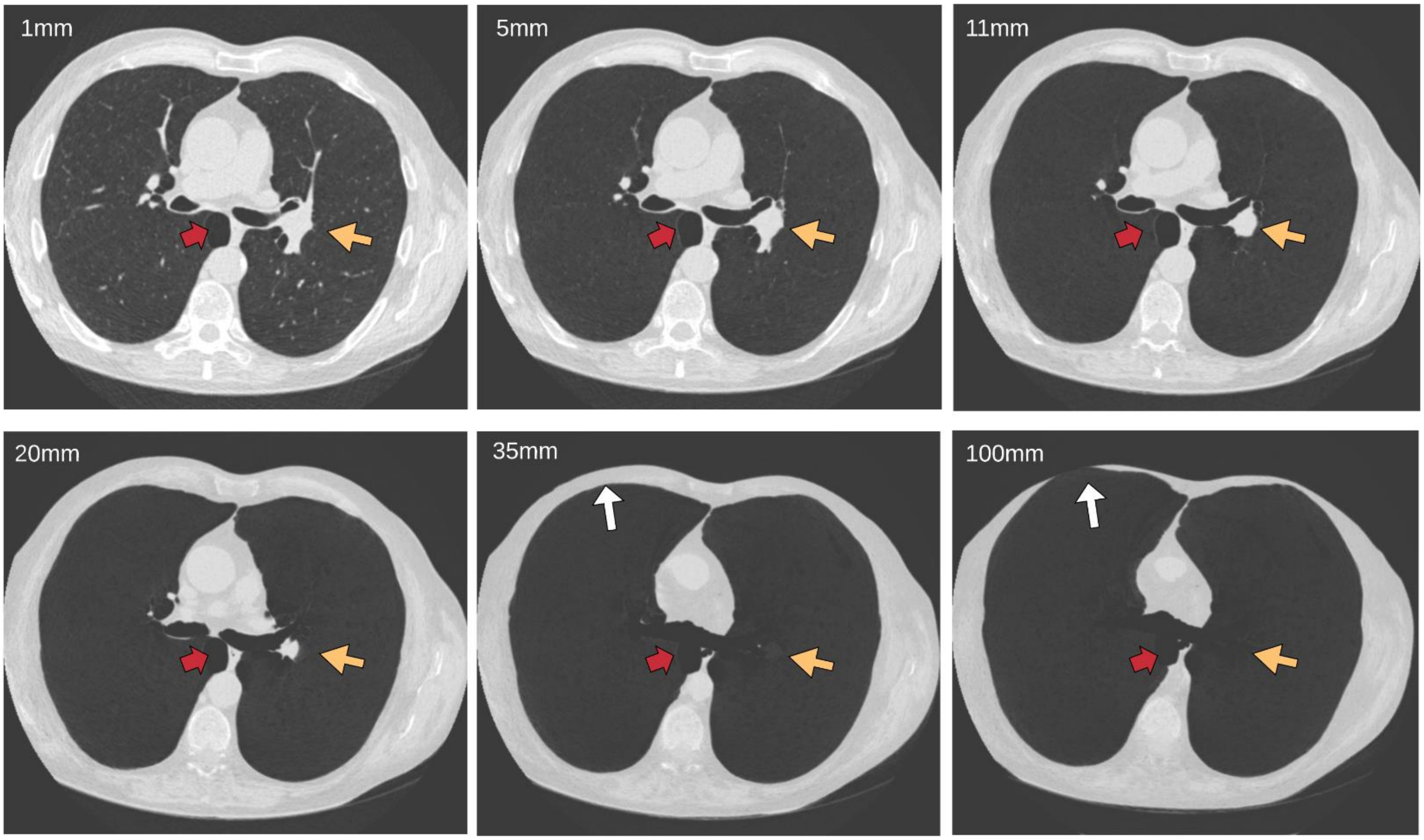

Fig. A: Illustration of emphysema case with different MinIP settings with slab thickness varying between 1mm to 100 mm. The arrow mark shows a region of emphysema around the around airways. We see that minIP with 1mm, 5 mm and 11 mm had better visualisation of parenchyma regions by removing high contrast regions like vessels. However, in the higher minIP starting from 20 mm , 35 mm and 100 mm the emphysema regions merges with

airways (the other low attenuation regions) leading to loss of structural information.

Online Resource 2: Class separation plots were generated from the external validation, based on the anomaly score for the models with different minIP settings and without minIP. To evaluate the 95% confidence interval (CI) and to visualise the variation of statistics for each setting, empirical bootstrap resampling (with 1000 bootstrap replications of statistic mean) was applied. The class separation plots of 3 different minIP settings and without minIP is shown below. With minIP the percentage overlap was smaller (15%) when compared to without minIP (31%), indicating a better class separation.

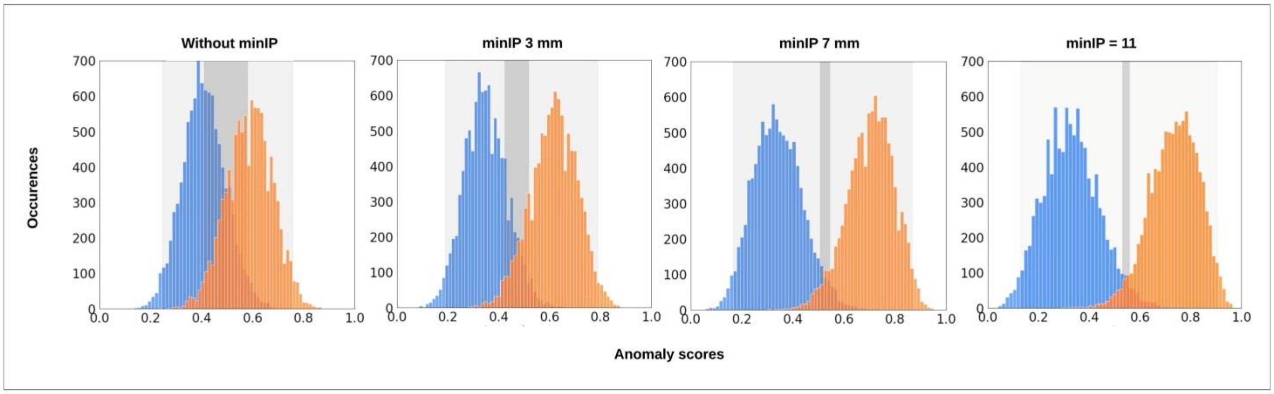

Fig. B: Illustrates class separation plots of with and without minIP (bootstrap resampling applied). Where blue colour represents the non-emphysematous, red represents the emphysematous scans, and grey blocks represent the 95% CI. The dark grey indicates the overlap regions between normals and abnormal scans on internal validation using descriptive statistics considering the distribution as normal.
